# Optimising genetic transformation of *Trypanosoma cruzi* using hydroxyurea-induced cell-cycle synchronisation

**DOI:** 10.1016/j.molbiopara.2018.07.002

**Published:** 2018-12

**Authors:** Francisco Olmo, Fernanda C. Costa, Gurdip Singh Mann, Martin C. Taylor, John M. Kelly

**Affiliations:** a Department of Pathogen Molecular Biology, London School of Hygiene and Tropical Medicine, Keppel Street, London, WC1E 7HT, UK; b Institute of Physics of São Carlos, University of São Paulo, São Carlos, 13563-120, Brazil

**Keywords:** *Trypanosoma cruzi*, Electroporation, Transfection efficiency, Hydroxyurea

## Abstract

The limited flexibility and time-consuming nature of the genetic manipulation procedures applicable to *Trypanosoma cruzi* continue to restrict the functional dissection of this parasite. We hypothesised that transformation efficiency could be enhanced if electroporation was timed to coincide with DNA replication. To test this, we generated epimastigote cultures enriched at the G1/S boundary using hydroxyurea-induced cell-cycle synchronisation, and then electroporated parasites at various time points after release from the cell-cycle block. We found a significant increase in transformation efficiency, with both episomal and integrative constructs, when cultures were electroporated 1 h after hydroxyurea removal. It was possible to generate genetically modified populations in less than 2 weeks, compared to the normal 4–6 weeks, with a 5 to 8-fold increase in the number of stably transformed clones. This straightforward optimisation step can be widely applied and should help streamline functional studies in *T. cruzi.*

## Highlights

• A straightforward method for optimising *Trypanosoma cruzi* transfection efficiency.
• Facilitated by hydroxyurea-induced cell-cycle synchronization.
• Applicable to both episomal and integrative-mediated transformation.
• Reduces the time required to generate genetically modified cell lines.
• Increases the number of stably transformed clones.

The protozoan parasite *Trypanosoma cruzi* is the causative agent of Chagas’ disease, an infection endemic throughout Latin America, causing high levels of morbidity and mortality. There is no vaccine, and the available drugs can have toxic side-effects, with treatment failures widely reported. The completion of the *T. cruzi* genome project provided a major opportunity to gain new insights into parasite biology, disease pathogenesis and resistance mechanisms, as well as providing an improved framework for drug and vaccine development. Although there has been significant progress, the time-consuming nature of *T. cruzi* genetic manipulation procedures has been a rate-limiting step [1]. Advances have also been restricted by the absence of RNAi machinery, which in *Trypanosoma brucei*, has been exploited to dissect drug-resistance mechanisms at a genome-wide level [2,3]. However, recent reports on the transfer of CRISPR/cas9 technology to *T. cruzi* [[4], [5], [6]], and the possibility of using forward genetics by constructing over-expression libraries, suggests that high-throughput functional screening approaches might be applicable to this parasite, if transfection efficiency can be improved. Electroporation of epimastigotes is the only transfection method currently used for genetic modification of *T. cruzi* [1]. However, it is relatively inefficient and the process takes 4–6 weeks to produce an initial population of transformants. This time-scale is significantly lengthened when the aim is to generate null mutants, since two rounds of transfection have to be undertaken, plus an additional round when a rescue vector is required [7]. Here we describe a simple optimization step that enhances *T. cruzi* transfection efficiency, resulting in an increase in the number of transformants generated and a significant reduction in the time required to select a drug-resistant population.

**Fig. 1:**
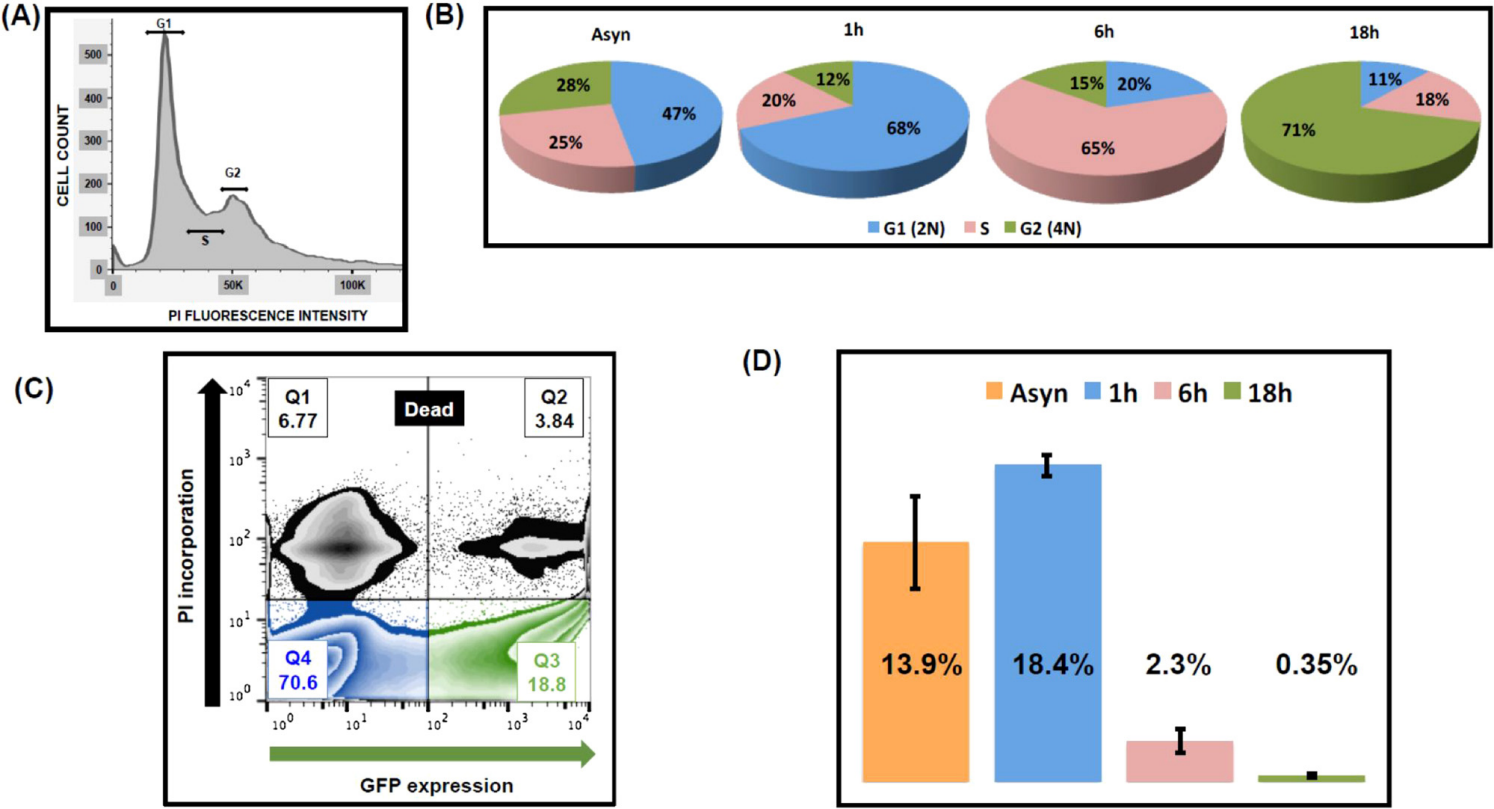
Transient transfection of *Trypanosoma cruzi* following hydroxyurea (HU) treatment. Exponentially growing *T. cruzi* (CL Brener strain) epimastigotes were treated with 20 mM HU for 24 h, washed twice with PBS and then re-suspended in fresh growth medium at 28 °C [14]. Flow cytometry was then used to assess the cycle-cycle status of the population. (A). Representative FACS histogram (non-treated population) showing the number of cells in each cell-cycle stage, inferred by measuring the DNA content using propidium iodide (PI) staining and a BD LSR II Flow Cytometer. (B). Percentage of parasites in the G1, S and G2 stages of the cell-cycle, 1 h, 6 h and 18 h after HU removal, with an asynchronous culture (Asyn) as the control. (C) 2 × 10^7^ epimastigotes were electroporated in the presence of 5 μg pTREXn-GFP, following incubation in the presence of 20 mM HU, with a control non-synchronized population (see text). After 48 h incubation in growth medium, parasites were assessed using a BD FACSCalibur™, with PI incorporation and GFP expression. Gating was adjusted using live wild-type parasites and paraformaldehyde-fixed wild-type parasites (data not shown). Parasites in the PI-ve/GFP + ve window were judged to be the transiently transfected population. (D) Percentage of live parasites in the population that express GFP 48 h after electroporation, as determined by FACS. Data are derived from triplicate experiments.

To explore the effect of the cell-cycle phase on transfection efficiency, we first induced exponentially growing *T. cruzi* epimastigotes to arrest at the G1/S boundary by incubation with 20 mM hydroxyurea (HU) for 24 h. HU inhibits ribonucleotide reductase, causing depletion of dNTPs and inhibition of DNA replication [[8], [9], [10], [11]]. Following HU removal, the parasite population was assessed by flow cytometry (Fig. 1A). Within 6 h, the majority of cells had transitioned from G1 to S phase, and by 18 h, most had progressed to G2 (Fig. 1B). Epimastigotes were electroporated with the episomal vector pTREXn-GFP [12] at various time points after release from the cell-cycle block. We used the Amaxa Nucleofector (programme X-014) with buffer Tb-BSF [13], conditions we had found to be optimal for *T. cruzi*. After electroporation, parasites were transferred to 10 ml of fresh medium [14] and assessed by flow cytometry 48 h later. Transient transfection efficiency (assessed on the basis of GFP expression) (Fig. 1C and D) was highest in the parasites electroporated 1 h after the removal of HU (18.4 + 0.6%), followed by the non-treated population (13.9 + 2.7%) (*p* = 0.005), and then those electroporated 6 h (2.3 ± 0.7%) and 18 h (0.4 ± 0.1%) after the removal of HU (Fig. 1E).

**Fig. 2:**
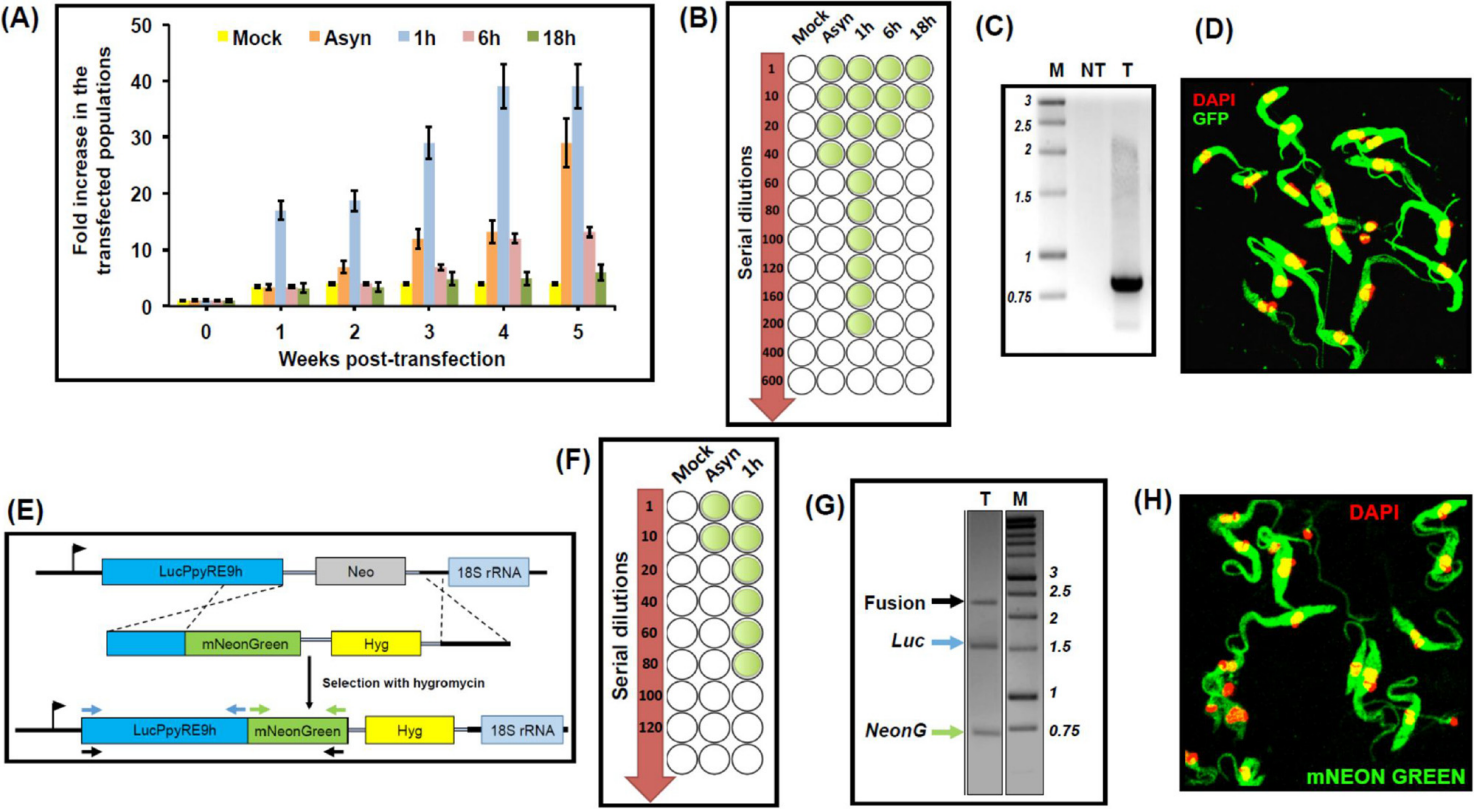
Effect of hydroxyurea (HU) treatment on the generation of stably transformed *Trypanosoma cruzi* following transfection with episomal and integrative vectors. (A) Outgrowth of G418-resistant (100 μg/ml) *T. cruzi* epimastigotes following incubation in 20 mM HU for 24 h, and transfection with pTREXn-GFP (legend to Fig. 1) at various time points after release from the cell-cycle block. Parasite proliferation, determined at weekly intervals, is presented as the fold increase compared to the number of cells transfected (experiment in triplicate). (B) Assessing episomal transformation efficiency by limiting dilution. Following transfection with pTREXn-GFP, HU treated parasites were subjected to serial dilution under selective pressure (100 μg/ml G418) in 48 well plates (total volume, 1 ml per well). The minimum number of drug-resistant parasites generated per μg of episomal DNA can be inferred from the wells (highlighted in green) containing drug-resistant parasites 8 weeks post-transfection (performed in triplicate). (C) DNA was prepared from transfected (T) and non-transfected (NT) parasites and assessed by PCR using primers specific to the neomycin phosphotransferase (Neo) gene. (D) Epimastigotes were fixed in 2% paraformaldehyde and imaged on a Zeiss LSM 510 confocal microscope to identify those expressing GFP (green). DNA is stained with DAPI (red). (E) Construct used for integrative transformation. Sequences derived from the 3′-end of the red-shifted luciferase gene *LucPpyRE9h* and the region upstream of an 18S rRNA gene were arranged to facilitate targeted integration [6], generating a bioluminescent:fluorescent (mNeonGreen) fusion protein gene and conferring hygromycin (Hyg) resistance. (F) Assessing integrative transformation by limiting dilution. Following transfection, parasites were subjected to serial dilution under selective pressure (100 μg/ml hygromycin). The number of parasites generated per μg of DNA (performed in triplicate) can be inferred as outlined above. (G) Confirmation of integration using PCR. Primer pairs are colour-coded (see image 2E). (H) Transformed epimastigotes (23 days post-transfection cultured under continuous hygromycin-selection) imaged by confocal microscopy showing acquisition of fluorescence (green). DNA is stained with DAPI (red).

Following addition of the selective drug G418 (100 μg/ml), we monitored the outgrowth of stably transformed drug-resistant parasites. Under selective pressure, replication of mock-transfected parasites ceased within 7 days (Fig. 2A). Parasites electroporated 1 h after HU removal displayed rapid outgrowth of drug-resistant transformants, and were suitable for sub-culturing and expansion within 14 days, a situation not achieved until week 5 in the case of the non-synchronised population (Fig. 2A). With parasites transfected 6 and 18 h after HU removal, the outgrowth of stable transformants took up to 8 weeks. To assess the efficiency of stable transformation, parasites transfected with pTREXn-GFP were plated by limiting dilution under G418-selection. At least 200 transformants/μg of DNA were generated when parasites were transfected 1 h after release from the cell-cycle block, five times the number achievable with asynchronised cells (Fig. 2B–D). To investigate the effect on targeted integration, we used the *T. cruzi* cell line *PpyRE9h* [15], which contains a luciferase gene inserted into a ribosomal locus (Fig. 2E). These parasites were transfected with a linear DNA construct designed to integrate at this site to create a chimeric bioluminescent/fluorescent protein gene and to replace the neomycin phosphotransferase gene with one that confers resistance to hygromycin [6]. When electroporation was carried out 1 h after HU removal, we found an eight-fold increase in the number of hygromycin-resistant transformants, compared to the numbers achievable with asynchronous cells (Fig. 2F). We were able to demonstrate that transfection had resulted in targeted insertion, expression of a fusion protein reporter and that the parasite population was universally fluorescent (Fig. 2G and H).

The HU-mediated cell-cycle synchronisation step improves the outcome of *T. cruzi* transfection experiments in two ways; first, by increasing the number of transformants generated, and second, by reducing significantly the time required to select an exponentially growing genetically modified population. HU treatment is known to result in stalled replication forks [16], which can lead to double-strand breaks (DSBs) and increased recombination during S phase [17]. Under the synchronisation conditions used (1 h, 20 mM; Fig. 1), no detrimental effects that were attributable to HU-mediated toxicity were observed, although we cannot exclude it as a possibility in some instances. In yeast, HU-treatment has been shown to promote targeted integration [18]. In *T. cruzi*, repair of DSBs by non-homologous end-joining is absent, with homologous recombination being the dominant process involved [4]. Therefore, the enhanced targeted integration that we observe (Fig. 2E–H) might result from DSBs induced by HU treatment. In the case of non-integrative transformation, the entry of episomal DNA into parasites as they are about to transition into S-phase could allow rapid replication and enhanced stability. At 6 h post-treatment however, when the majority of parasites will have completed genomic DNA replication [19], and be on the verge of entry into G2, this effect would be greatly reduced, with a concomitant drop in transfection efficiency. Whatever the precise mechanism, the procedures described here should help to streamline functional studies in *T. cruzi*, and may be more widely applicable to other protozoa.

## Declarations of interest

None.
